# Protozoan predation enhances stress resistance and antibiotic tolerance in *Burkholderia cenocepacia* by triggering the SOS response

**DOI:** 10.1093/ismejo/wrae014

**Published:** 2024-01-28

**Authors:** Álvaro Morón, Alaa E Tarhouchi, Iván Belinchón, Juan M Valenzuela, Patricia de Francisco, Ana Martín-González, Francisco Amaro

**Affiliations:** Department of Genetics, Physiology and Microbiology. Faculty of Biological Sciences, Complutense University of MadridMadrid 28040, Spain; Department of Genetics, Physiology and Microbiology. Faculty of Biological Sciences, Complutense University of MadridMadrid 28040, Spain; Department of Genetics, Physiology and Microbiology. Faculty of Biological Sciences, Complutense University of MadridMadrid 28040, Spain; Department of Genetics, Physiology and Microbiology. Faculty of Biological Sciences, Complutense University of MadridMadrid 28040, Spain; Department of Genetics, Physiology and Microbiology. Faculty of Biological Sciences, Complutense University of MadridMadrid 28040, Spain; Department of Genetics, Physiology and Microbiology. Faculty of Biological Sciences, Complutense University of MadridMadrid 28040, Spain; Department of Genetics, Physiology and Microbiology. Faculty of Biological Sciences, Complutense University of MadridMadrid 28040, Spain

**Keywords:** Burkholderia, persister, stress, SOS response, Tetrahymena, ciliates, protist, predation, ROS, antibiotic

## Abstract

Bacterivorous protists are thought to serve as training grounds for bacterial pathogens by subjecting them to the same hostile conditions that they will encounter in the human host. Bacteria that survive intracellular digestion exhibit enhanced virulence and stress resistance after successful passage through protozoa but the underlying mechanisms are unknown. Here we show that the opportunistic pathogen *Burkholderia cenocepacia* survives phagocytosis by ciliates found in domestic and hospital sink drains, and viable bacteria are expelled packaged in respirable membrane vesicles with enhanced resistance to oxidative stress, desiccation, and antibiotics, thereby contributing to pathogen dissemination in the environment. Reactive oxygen species generated within the protozoan phagosome promote the formation of persisters tolerant to ciprofloxacin by activating the bacterial SOS response. In addition, we show that genes encoding antioxidant enzymes are upregulated during passage through ciliates increasing bacterial resistance to oxidative radicals. We prove that suppression of the SOS response impairs bacterial intracellular survival and persister formation within protists. This study highlights the significance of protozoan food vacuoles as niches that foster bacterial adaptation in natural and built environments and suggests that persister switch within phagosomes may be a widespread phenomenon in bacteria surviving intracellular digestion.

## Introduction

Protozoan predation represents a major selection pressure that bacteria face in both natural and anthropogenic environments [1, 2]. Bacterivorous protozoa (traditionally termed as protozoa), such as ciliates, amoebae, and flagellates, tightly regulate bacterial populations, but may also act as “Trojan horses” serving as reservoir where pathogenic bacteria can survive and even replicate [3, 4]. Macrophages and protozoa kill bacteria by taking them up into phagosomes that become acidified and filled with reactive oxygen and nitrogen species (ROS/RNS) and hydrolytic enzymes. During phagocytosis, ROS (such as superoxide and hydrogen peroxide) and RNS (such as nitric oxide) are produced by phagosomal NADPH oxidase complex (NOX) and the cytosolic inducible nitric oxide synthase (iNOS), respectively, in a process called oxidative burst or respiratory burst [5]. Genes encoding NOX and iNOS homologs have been found in the genomes of some protists, suggesting that this process also operates in the protozoan phagosome [6–8]. However, some bacterial species can evade intracellular digestion by employing different mechanisms [9], subsequently infecting their predators [10, 11], or are eventually released packaged in vesicles expelled by protists which are often named fecal pellets or expelled food vacuoles (EFVs) [12–15]. Interestingly, studies have shown that pathogenic bacteria released from protozoan food vacuoles exhibit enhanced resistance to harsh conditions, antibiotics and even increased virulence [15–17], suggesting that EFVs may act as vehicles for pathogen transmission. In fact, the size of EFVs (2–6 μm in diameter) falls within the range of respirable particles [14], and thus may reach the lung alveoli leading to infection, although this has not been demonstrated yet. Furthermore, in a seminal work, Espinoza-Vergara *et al*. [15] showed that *Vibrio cholerae* released from EFVs exhibited colonization advantages in the mouse digestive tract. The molecular mechanisms underlying bacterial packaging into EFVs remain to be elucidated. While it is believed to be a protozoan-driven process, bacterial virulence factors appear to play an important role [14, 15, 18].

The opportunistic pathogen *Burkholderia cenocepacia* is a member of the *B. cepacia* complex (Bcc), a group of 24 closely related bacterial species known to cause life-threatening infections in cystic fibrosis (CF) and chronic granulomatous disease (CGD) patients [19, 20]. Moreover, Bcc bacteria have also been associated with nosocomial infections in immunocompromised and immunocompetent patients hospitalized for reasons unrelated to CF and CGD [21]. Currently, *B. cenocepacia* and *B. multivorans* are the Bcc species most frequently isolated from patients in Europe and North America [22]. *B. cenocepacia* is multidrug resistant and transmissible among CF patients, and causes a fatal condition known as “cepacia syndrome” which is characterized by necrotizing pneumonia and progressive respiratory failure. In particular, *B. cenocepacia* strain K56-2 is a member of the highly transmissible epidemic lineage ET12 that is responsible for most outbreaks [23]. The isolation of clonal strains of *B. cenocepacia* from both infected patients and the environment suggests that the latter is a potential reservoir for infection in the absence of nosocomial or patient-to-patient transmission [24, 25]. *B. cenocepacia* survives within mammalian macrophages [26] and human airway epithelial cells [27]. However, little is known about its interactions with non-human hosts. In vitro*, B. cenocepacia* has been shown to kill the nematode *Caenorhabditis elegans* [28], and is able to survive without replicating inside acidic vacuoles of the free-living amoeba *Acanthamoeba polyphaga* [29]. Thus, although amoebae were proposed to serve as reservoirs for Bcc bacteria in the environment [30], the role that protozoa play in the epidemiology and ecology of Bcc is currently unknown. To date, no study has investigated whether Bcc bacteria can be packaged into EFVs by protists. Hence, as ciliates and Bcc bacteria co-occur in the same ecological niches [1], and given the likely implication of protozoan EFVs in pathogen dissemination, we aimed to better understand the interaction between ciliates and *B. cenocepacia*. By gaining insights into Burkolderia–ciliates interactions, we hope to identify potential pathways for controlling the spread of Bcc bacteria and reducing the risk of infections associated with them.

Here, we report that ciliates isolated from hospital and domestic sink drains expel EFVs laden with viable *B. cenocepacia* bacteria. Passage through ciliates enhanced stress resistance and produced antibiotic-tolerant persister cells in *B. cenocepacia*. Persisters are defined as subpopulations of bacterial cultures that transiently adopt a phenotype characterized by a non-growing state tolerant to lethal concentrations of antibiotics [31]. They are a major contributor to treatment failure and recalcitrance of chronic infections as those in CF patients. Besides stochastic generation in bacterial populations [32], environmental stress is believed to trigger persister formation as well [31, 33]. While persister cells have been identified in all major pathogens, only a few studies have investigated the switch to the persister phenotype within eukaryotic cells [34–36]. We show here that SOS activation by ROS is required for persister formation within the protozoan phagosome, further revealing that ciliate food vacuoles are genuine hot spots for the generation of antibiotic-tolerant persisters in natural and built environments.

## Materials and methods

### Bacterial and protozoan strains and growth conditions

The bacterial strains and plasmids used in this study are listed in Tables S1 and S2, respectively. Strains of *B. cenocepacia* and *Escherichia coli* were grown in Lysogeny broth medium (LB) at 37°C. When required, antibiotics were added to the following final concentrations: 100 μg/ml trimethoprim (Panreac) for *B. cenocepacia* and 50 μg/ml for *E. coli*, 150 μg/ml tetracycline (Panreac) for *B. cenocepacia* and 20 μg/ml for *E. coli*, 100 μg/ml chloramphenicol (Panreac) for *B. cenocepacia* and 20 μg/ml for *E. coli*, and 40 μg/ml kanamycin (Panreac) for *E. coli*. Gentamicin (Panreac) was used at 50 μg/ml in conjugation assays to select against donor and helper *E. coli* strains.

*Tetrahymena elliotti* strain 4EA was cultured at 28°C in 2% proteose peptone (Pronadisa) supplemented with 10 μM FeCl_3_ (Sigma Aldrich) and 250 μg/ml of both streptomycin sulfate and penicillin G (Sigma Aldrich). *Colpoda* sp strain CSE36 and *Tetrahymena* sp strain T2305B2 were routinely cultured at 28°C in Page’s saline buffer [37] with heat-killed inactivated *E. coli* K-12 as food source.

Construction of all reporter and mutant strains employed in this study is described in the supplementary material.

### Assessment of *B. cenocepacia* resistance to protozoan predation

Bacterial intracellular survival was assessed by quantifying *B. cenocepacia* survival in co-culture with the protozoan predator. Given the high feeding rate of *Tetrahymena* species [38] a 1:200 protist:bacteria ratio was used. Synchronized log-phase cultures of bacteria (OD_600_ = 0.3) and ciliates (3×10^5^ cells/ml) were washed and resuspended in 0.01 M Tris–HCl pH 7.5 buffer. Co-cultures were established by combining protist and bacteria (1:200) in a 96-well plate for up to 48 h at 28°C. Every 24 h, intracellular bacteria were released by lysing protozoan cells with 1% Triton X-100 (Sigma Aldrich). The number of viable bacteria was quantified by colony forming unit (CFU) counts on LB agar plates. For each assay, at least three independent experiments were performed. Bacteria incubated in Tris–HCl buffer for up to 48 h were used as control. Addition of 1% Triton X-100 did not impair bacterial viability. Additionally, the Live/Dead BacLight Bacterial Viability kit (Themo Fisher Scientific) was used to assess the proportion of viable bacterial cells packaged within EFVs. For that purpose, co-cultures, set up as described above, were incubated for 15 min with the Live/Dead BacLight Bacterial Viability solution (Thermo Scientific) according to manufacturer’s directions. Cells and EFVs were then washed twice with 0.01 M Tris–HCl pH 7.5 and observed under an Olympus CX41 fluorescence microscope using a double-band filter set (λ_exc_ = 480–495/550–570 nm, λ_emi_ = 510–535/590–621 nm).

### Purification of EFVs containing *B. cenocepacia*

Bacteria–protist co-cultures were transferred to a sterile 50 ml conical tube to allow EFVs to aggregate and settle at the bottom. Then, the decanted EFVs were carefully transferred with a Pasteur pipette into a new sterile tube and centrifuged at 800× g for 5 min. Ciliates were allowed to swim back into suspension before removing the supernatant. The EFV pellet was then washed with 1 ml 0.01 M Tris–HCl pH 7.5 buffer. This step was repeated three-five times and observed under the microscope to check for the absence of ciliates. When required, bacteria were released from EFVs by adding 1% Triton-X100, harvested by centrifugation at 3500× g, washed and suspended in 0.01 M Tris–HCl pH 7.5. Examples of representative microscope images showing purified EFVs are shown in Fig. S1.

### Transmission electron microscopy

Samples from *B. cenocepacia* and *T. elliotti* co-cultures were fixed for 1 h in 0.1 M sodium cacodylate buffer pH 7.2 (TAAB Laboratories Equipment Ltd) containing 2.5% glutaraldehyde (Sigma Aldrich). Cells were then washed three times in cacodylate buffer and fixed with 0.5% OsO_4_ (TAAB Laboratories Equipment Ltd) in cacodylate buffer for 45 min at 4°C. Fixed cells were stained for 1 h with 1% uranyl acetate (TAAB laboratories Equipment Ltd), and later dehydrated in an acetone series of increasing concentration (24%, 50%, 75%, and 100%). After that, cells were embedded in Embed 812 resin (TAAB Laboratories Equipment Ltd) by following manufacturer’s directions. Ultrathin sections were obtained with microtome Ultracut (Reichert-Jung), stained with 2% uranyl acetate and lead citrate and observed in a JEM1010 transmission electron microscope at 80 kV at the Spanish National Centre for Electron Microscopy-Complutense University of Madrid.

### Detection of ROS production within protozoan phagosomes

ROS production within protozoan food vacuoles was assessed by staining cells with two different cell-permeable ROS fluorogenic probes: CellROX Orange Reagent (Molecular Probes) and 2′,7′-dichlorofluorescein diacetate (DCFH-DA) (Sigma Aldrich). Upon oxidation by ROS, CellROX Orange exhibits strong fluorescence with absorption/emission maxima of 554/570 nm. On the other hand, cytosolic cellular esterases convert DCFH-DA into DCFH, which upon oxidation turns into the green fluorescent DCF with absorption/emission maxima of 504/525 nm. In different assays log-phase bacteria were stained with 10 μM CellROX Orange or 10 μM DCFH-DA for 30 min, washed twice and resuspended in 0.01 M Tris–HCl pH 7.5. Then, stained bacteria were fed to ciliates using a 1:200 protist:bacteria ratio and transferred to a 96 well black plate (Thermo Scientific). Emission of CellROX Orange or DCF fluorescence was monitored automatically with a TECAN Infinite MPlex (Tecan Group Ltd) plate reader every 15 min. The excitation/emission wavelengths of CellROX Orange and DCF were 535–545/560–580 nm and 480–490/522–542 nm, respectively. In both cases, the fluorescence signal was normalized to OD_450_. In addition, ROS production within protozoan food vacuoles was visualized by feeding green fluorescent protein (GFP) expressing bacteria to 10 μM CellROX Orange-stained *T. elliotti* cells, or by feeding dsRed-expressing bacteria to 10 μM DCFH-DA-stained *T. elliotti*. After 1 h of co-incubation at 28°C, ciliates (and ingested bacteria) were harvested, washed twice with 0.01 M Tris–HCl pH 7.5 and observed under an Olympus CX41 fluorescence microscope using a blue excitation filter (λ_exc_ = 470–495 nm, λ_emi_ = 510–535 nm), a green excitation filter (λ_exc_ = 530–550 nm, λ_emi_ = 570–590 nm), and a double-band filter set (λ_exc_ = 480–495/550–570 nm, λ_emi_ = 510–535/590–621 nm). Fluorescence microscopy images were analyzed with ImageJ software [39] to estimate the percentage of food vacuoles that exhibited both CellROX Orange and GFP fluorescence.

### P*_recA_-egfp* and P*_katB_-egfp* reporter assays

Co-cultures protist:bacteria were established in 0.01 M Tris–HCl pH 7.5 as described above and transferred to a 96 well black plate (Thermo Scientific). GFP fluorescence was monitored automatically by measuring green fluorescence with 480–490 nm excitation and 522–542 nm emission wavelengths nm with a TECAN Infinite MPlex plate reader every 30 min for 20 h at 28°C. The fluorescence signal was normalized to OD_450_.

### RNA extraction and quantitative reverse transcription PCR (qRT-PCR)

Co-cultures protist:bacteria (1:200) were established in 0.01 M Tris–HCl pH 7.5 as described above and incubated at 28°C for 3–24 h. At 3 and 24 h of co-incubation, RNA from EFV-encased *B. cenocepacia* was isolated by lysing ciliates and EFVs on ice for 20 min in an RNA stabilization solution (1% acidic phenol from Fisher Scientific, 19% ethanol in DEPC-H_2_O_2_ from Sigma Aldrich) [40] supplemented with 0.1% SDS (Fisher Scientific). Released bacteria were then collected by centrifugation (3500× g, 15 min) and total RNA was extracted and DNAseI-treated using the Direct-zol RNA miniprep plus kit (Zymo Research). As control, total RNA was also isolated from bacteria incubated for 3 and 24 h in 0.01 M Tris–HCl pH 7.5 in the absence of ciliates by following the same procedure.

*T. elliotti* RNA was isolated from bacteria-ciliate co-cultures as follows. At each time point (1, 4, and 6 h), ciliates were harvested by centrifugation (1300× g, 2 min) and incubated in RNA stabilization solution (1% acidic phenol, 19% ethanol) on ice for 20 min. Then, ciliates were harvested by centrifugation and total RNA was isolated and DNAseI-treated with the Direct-zol RNA miniprep plus kit. As control, RNA was also extracted from ciliates incubated for 1, 4 and 6 h in in 0.01 M Tris–HCl pH 7.5 in the absence of bacteria by following the same procedure.

RNA quality was verified by 1% agarose electrophoresis and quantified using Nanodrop One (Thermo Scientific). Isolated RNA was retrotranscribed with the Maxima Reverse Transcriptase (Thermo Scientific) by following manufacturer’s directions. cDNA samples were then amplified in duplicate in 96 microtiter plates (4titude). Real time PCR was carried out in 20 μl reaction mixtures containing 1× TB Green Premix Ex Taq (Takara), 1x ROX reference dye (Takara), 300 nM of each forward and reverse primer (Integrated DNA Technologies) and 2 μl (25 ng) of the template cDNA. Samples were amplified in a iQ5 real-time PCR (Bio-Rad) using the following thermal cycling protocol: 5 min at 95°C, followed by 40 cycles of 30 sec at 95°C, 30 sec at 55°C and 20 sec at 72°C; and a final step of 1 min at 95°C and 1 min at 55°C. The specificity of each primer pair was confirmed by melting curve analysis. The fold change in mRNA expression levels was calculated with the 2^-ΔΔCt^ method [41] and normalized against the expression level of the housekeeping gene *gyrA* (locus tag K562_12858, accession number NZ_CP053300) or *ACT-1* (locus tag EI7_07658.1, Genome accession number PRJNA262690). Non-template controls (NTC) and RT minus controls were negative. Primers used in real time PCR reactions are shown in Table S3.

### Assessment of stress resistance of *B. cenocepacia* recovered from protists and persister assays

Co-cultures protist:bacteria (ratio 1:200) were established in 0.01 M Tris–HCl pH 7.5 as described above. As control, since this buffer may add nutritional stress to *B. cenocepacia*, the same number of log-phase bacteria was incubated in buffer in the absence of ciliates. After 24 h, EFVs were collected from co-cultures and washed twice with 0.01 M Tris–HCl pH 7.5. Bacteria were then released by adding 1% Triton-X100 and pelleted by centrifugation at 3500× g for 5 min. Released bacteria were washed twice with 0.01 M Tris–HCl pH 7.5. In parallel, control bacteria incubated in 0.01 M Tris–HCl pH 7.5 for 24 h were harvested, exposed to 1% Triton-X100 and washed twice with 0.01 M Tris–HCl pH 7.5. At this point, both bacteria released from EFVs and control bacteria were subjected to different stress conditions (0.02% H_2_O_2_, starvation and desiccation) for the indicated time intervals. At selected time points bacterial suspensions were subsequently serially diluted 1:10 in 0.9% NaCl and plated on LB agar plates. The number of surviving bacteria was estimated by CFU counts. For each assay three independent experiments were performed. In desiccation resistance assays, bacteria were spotted onto cellulose filter disks and dried at 30°C for up to 24 h, then rehydrated in 0.9% NaCl for 30 min and spread on LB agar plates to allow for CFU counts.

For persister assays, bacteria (released from EFVs and controls prepared as described above) at 10^7^ CFU/ml were incubated in LB buffered medium supplemented or not (control) with 40–80 μg/ml (10–20× MIC) ciprofloxacin (Sigma Aldrich) or 320 μg/ml (10×MIC) meropenem (Sigma Aldrich) for up to 20 h at 37°C. At 0, 3 and 20 h, bacteria were harvested by centrifugation and washed in 10 mM MgSO_4_ (Sigma Aldrich) to inactivate the residual antibiotic. The bacterial suspensions were then serially diluted in 0.9% NaCl and plated on LB agar plates to quantify the number of surviving bacteria by CFU counting. Then, to confirm that surviving bacteria were true persisters, survivors of 20 h of antibiotic treatment were harvested from plates, grown overnight in fresh LB and used to perform a follow-up persister assay.

### Paraquat protection assay

Bacteria from synchronized log-phase cultures (OD_600_ = 0.3) were incubated in LB buffered medium supplemented or not (control) with 1 mM Paraquat (Sigma Aldrich) for 30 min at 37°C in a 12-well polystyrene plate. The number of viable bacteria after treatment (time = 0) was estimated by plating serial dilutions on LB agar plates and CFU counts. Then, 40 μg/ml ciprofloxacin was added to each well and bacteria were incubated with the antibiotic for up to 20 h at 37°C. At given time points, bacteria were collected from each well, washed in 10 mM MgSO_4_ to inactivate the residual antibiotic, and plated on LB agar to estimate the number of survivors by CFU counting.

### NOX inhibition assay and iNOS inhibition assay

For NOX inhibition assays, *T. elliotti* cells (5×10^5^ cell/ml) were pre-treated with 10 μM VAS2870 (Cayman Chemicals) for 1 h at 28°C before adding the bacteria. Then, bacteria from synchronized log-phase cultures (OD_600_ = 0.3) were added to pre-treated and untreated (control) ciliates and incubated for 3 h at 28°C. Afterwards, ciliates were lysed with 1% Triton X-100 and the released bacteria were washed and incubated at 37°C in fresh LB buffered medium supplemented with 40 μg/ml (10× MIC) ciprofloxacin in a 24-well plate. Three hours later, bacteria were collected from each well, washed in 10 mM MgSO_4_ and plated on LB agar to estimate the number of survivors by CFU counting. The percentage of survival after ciprofloxacin challenge was determined by comparing survivors after 3 h of antibiotic exposure to survivors of the corresponding untreated group at the same 3 h interval. iNOS inhibition assays were performed by following the same procedure but pre-treating *T. elliotti* with 100 μM 1400 W (Medchem Express) for 1 h at 28°C before adding the bacteria.

The P*_recA_-egfp* reporter assay was performed by incubating the *B. cenocepacia* strain FA161 with ciliates pre-treated for 1 h with 10 μM VAS2870 or 100 μM 1400 W and untreated ciliates (control) in a 24-well plate. After 3.5 h of co-incubation with bacteria, ciliates were harvested and lysed with 1% Triton-X100 to release ingested bacteria from food vacuoles. Aliquots (150 μl) from each sample were transferred to a 96 well black plate (Thermo Scientific) and GFP fluorescence was measured with a TECAN Infinite MPlex plate reader (480–490 nm excitation/522–542 nm emission filter). Samples were measured in triplicate. VAS2870-treated and 1400 W-treated ciliates incubated in the absence of bacteria, and bacteria incubated without ciliates were used as fluorescence controls. Three independent experiments were performed.

### Statistical analysis

Statistical analyses were performed with GraphPad Prism v.8 (GraphPad Software). Statistical differences were determined using unpaired Student’s t-test when comparing two conditions and one-way analysis of variance with Dunnett’s post-test when comparing three or more conditions. Details of statistical analysis performed for each set of data are shown in the figure captions.

## Results

### *B. cenocepacia* bacteria resist intracellular digestion in ciliates and are packaged into EFVs with enhanced tolerance towards stress and antibiotics

**Figure 1:**
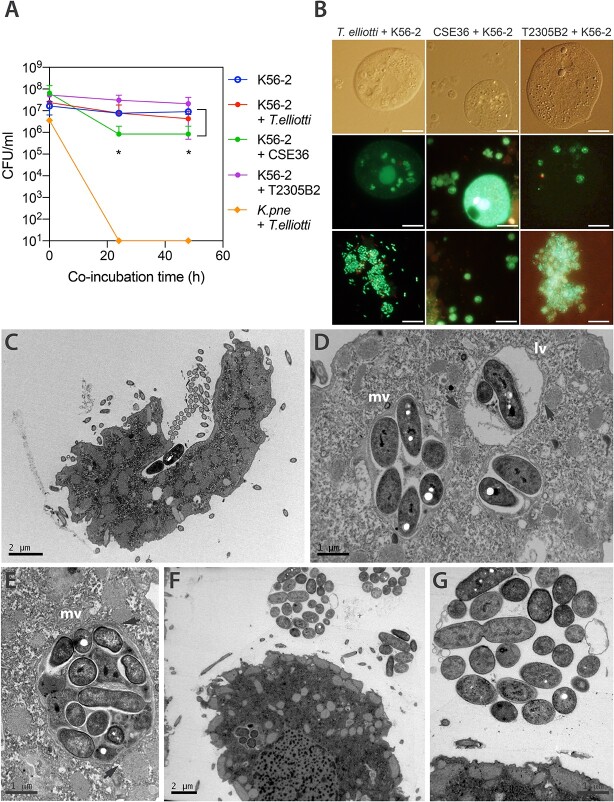
Grazing resistance of *B. cenocepacia* K56–2 and packaging of viable bacteria within EFVs by ciliates. (A) *B. cenocepacia* K56–2 survival under predation by wild (CSE36 and T2305B2) and domesticated (*T. elliotti*) ciliates. The number of surviving bacteria incubated in the absence (empty circles) or presence (filled circles) of ciliates was quantified by CFU counts after 0–48 h of co-incubation. The star (^*^) indicates significant differences (*P* < .05) between K56–2 + CSE36 vs K56–2 samples according the unpaired Student’s t-test. (B) Viability of ingested *B. cenocepacia* K56–2 and bacteria packaged inside EFVs produced by *T. elliotti* 4EA (pictures on the left), *Colpoda* sp CS36 (center) and *Tetrahymena* sp T2305B2 (right). Aliquots of the bacteria:ciliate co-cultures were stained after 24 h of co-incubation with the live/dead BacLight solution and observed with an epifluorescence microscope to visualize intact (green, Syto9-stained) or membrane-damaged (red, PI-stained) bacterial cells within intracellular food vacuoles and EFVs. Scale bar represents 10 μm. (C-G) TEM micrographs of *T. elliotti* grazing on *B. cenocepacia* K56–2. Bacteria being engulfed within a nascent food vacuole at the ciliated cytopharynx (C). Most K56–2 bacteria survived within mid-stage and late-stage food vacuoles (D, E) and were eventually packaged into EFVs and expelled by ciliates (F, G). Mid-stage (mv) food vacuoles are characterized by a membrane that closely molds to the shape of enclosed bacteria. In contrast, late-stage (lv) vacuoles are spherical and exhibit a distinctive space or halo between the enclosed bacteria and the smooth vacuole’s membrane. Arrowheads point at mitochondria located near the food vacuoles. Dividing bacteria were observed within EFVs (G).

Survival assays were performed to determine whether *B. cenocepacia* K56–2 was able to evade intracellular digestion and/or multiply within different ciliates. *B. cenocepacia* survived predation although we observed different degree of grazing resistance depending on the ciliate predator (Fig. 1 A). *Colpoda* sp CSE36 (wild strain isolated from domestic sink drain) caused a greater reduction (2 log_10_) in the K56–2 population, whereas *T. ellioti* 4EA (laboratory strain) and *Tetrahymena* sp T2305B2 (wild strain isolated from hospital sink drain) reduced it in less than 1 log_10_. Accordingly, fluorescence microscopy after Syto9/PI staining (Fig. 1, B) and TEM (Fig. 1, C-G) confirmed that most *B. cenocepacia* cells avoided intracellular digestion and viable bacteria, sometimes dividing, were packaged in membrane-bound vesicles that were eventually expelled by ciliates into the extracellular medium within 3 h post-feeding, similar to previously reported *Legionella pneumophila* or *V. cholerae* laden EFVs [13, 15]. Similar results were found when we observed EFVs produced by a wild *Tetrahymena* sp T2305B2 isolated from hospital sink drain (Fig. S2).

**Figure 2:**
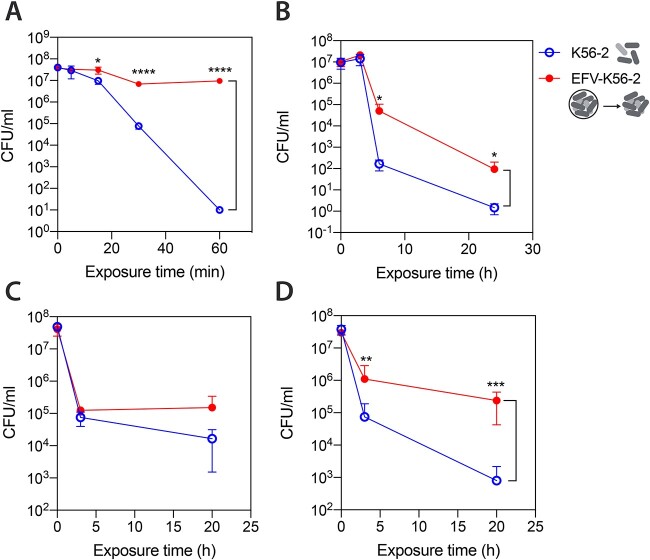
Increased stress resistance and antibiotic tolerance of *B. cenocepacia* K56–2 after passing through *T. elliotti*. (A-D) Survival rate of K56–2 cells released from EFVs (filled circles) and control planktonic K56–2 cells (empty circles) subjected to 0.02% H_2_O_2_ (A), desiccation (B), 320 μg/ml (10× MIC) meropenem (C), and 40 μg/ml (10× MIC) ciprofloxacin (D). At selected time points, bacteria were plated on LB and surviving bacteria were quantified by CFU counts. Data shown correspond to the average value from three independent biological replicates. Significant differences (^*^P < .05, ^*^^*^P < .01, ^*^^*^^*^P < .001, ^*^^*^^*^^*^P < .0001) between EFV-K56–2 and K56–2 (control) for each time point were determined using unpaired Student’s t-test.

Since passage through ciliates has been shown to increase environmental fitness in several grazing resistant bacteria [15–17], we assessed the stress resistance of *B. cenocepacia* K56–2 recovered from *T. elliotti* EFVs. Because we could not establish axenic cultures of undomesticated ciliates isolated from sink drain biofilms, we performed these experiments using *T. elliotti* 4EA as predator to avoid possible interferences with the indigenous bacterial species carried by the wild protists. We believe *T. elliotti* 4EA is a good surrogate model since we have isolated *Tetrahymena* species such as strain T2305B2 from hospital and domestic sink drains (Amaro, unpublished data). Thus, packaged K56–2 bacteria from *T. elliotti*-produced EFVs were released with 1% Triton X-100 and subsequently exposed to different stress conditions: 0.02% H_2_O_2_, starvation, desiccation, and antibiotics (ciprofloxacin, meropenem, tobramycin) at 10–20× MIC. As control, K56–2 cells incubated overnight in 0.01 M Tris–HCl pH 7.5 in the absence of ciliates were exposed to same conditions. Bacteria recovered from EFVs exhibited a higher resistance towards H_2_O_2_ exposure, surviving (0.5 log_10_ reduction, 26% survival after 60 min) at H_2_O_2_ doses (0.02%) that eradicated control bacteria in 60 min (6 log_10_ reduction, < 0.0001% survival) (Fig. 2 A). Additionally, EFV-released K56–2 bacteria survived desiccation better. After 6 h viability of control K56–2 decreased 5 log_10_, while viability of EFV-released bacteria dropped 2 log_10_. After 24 h, the viability of EFV-released bacteria was 1log_10_ higher than that of control bacteria (Fig. 2 B). However, we did not observe any protective effect towards starvation, as similar survival rates were observed for control and EFV-released bacteria (data not shown). Bacteria recovered from EFVs exhibited a higher tolerance towards high concentrations (10–20× MIC) of different classes of antibiotics. Passage through *T. elliotti* produced a 10 and > 100-fold increase in the proportion of bacteria tolerant to meropenem (a carbapenem, beta-lactam that blocks peptidoglycan biosynthesis) and ciprofloxacin (a quinolone, DNA-gyrase inhibitor), respectively (Fig. 2 C-D and Fig. 5 E). Time-kill curves performed in the presence of high concentrations (10× MIC) of these antibiotics alone revealed a biphasic killing curve, suggesting that antibiotic-tolerant bacteria recovered from EFVs were persisters (Fig. 2 D). Accordingly, the progeny of bacteria that survived antibiotic challenge were as susceptible as the initial inoculum and similar biphasic killing curves were obtained in follow-up experiments (Fig. S3), indicating that they were “bona fide” persisters. However, bacteria released from protozoan EFVs were killed by >4× MIC tobramycin (data not shown), indicating that persisters generated within *T. elliotti* food vacuoles were susceptible to this aminoglycoside. Consistent with our results, susceptibility to aminoglycosides in persisters that are tolerant to quinolones and beta-lactams has been thoroughly documented [42–46] . Overall, these results show that successful passage through ciliates confer enhanced resistance to oxidative stress and desiccation as well as some of the antibiotics that are prescribed to treat *B. cenocepacia* infections [47, 48], which could favor pathogen dissemination in the environment (natural or man-made) and colonization of the human host.

### ROS generated within *T. elliotti* phagosomes trigger the SOS response in *B. cenocepacia* and promote antibiotic tolerance

**Figure 3:**
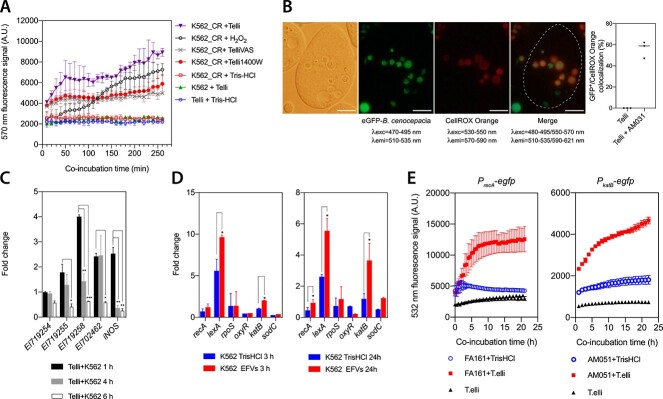
ROS production and upregulation of *B. cenocepacia* stress-related genes in protozoan phagosomes*.* (A) Quantification of ROS production as red (570 nm) fluorescence emitted by CellROX-stained *B. cenocepacia* K56–2 ingested by *T. elliotti*. A dramatic increase in the production of red fluorescence was detected for CellROX-stained K56–2 cells that were incubated in the presence of *T. elliotti* (inverted triangle) or 0.02% H_2_O_2_ (empty circle, positive control). No fluorescence signal was detected in CellROX-stained bacteria incubated in the absence of ciliates (filled square). Treatment of ciliates with inhibitors of NOX (VAS2870, cross) or iNOS (1400 W, circle) significantly decreased the fluorescence signal, supporting that CellROX was oxidized by protozoan-derived ROS. (B) CellROX Orange fluorescence co-localized with 55% of the food vacuoles containing GFP-expressing bacteria, confirming ROS exposure. Scale bar represents 10 μm. (C) Overexpression of *T. elliotti* genes encoding NOX (*EI71954, EI719255, EI719258, EI702462*) and iNOS homologs at 1–6 h post-feeding with *B. eenocepacia*. The rapid upregulation of genes encoding iNOS and NOX also confirmed that *T. elliotti* was feeding phagotrophically on bacteria. (D) Upregulation of SOS response and oxidative stress related genes in *B. cenocepacia* K56–2 recovered from protozoan food vacuoles (at 3 and 24 h post-feeding). Significant differences (^*^P < .05) were determined with the unpaired Student’s t-test. (E) Time-course quantification of GFP fluorescence emitted by the reporter strains *B. cenocepacia* FA161 *(*P*_recA_*-*egfp*) and AM051 (P*_katB_*-*egfp*) incubated with (square) or without (circle) *T. elliotti*. The dramatic increase in GFP fluorescence detected in the presence of ciliates suggests the upregulation of the SOS response and *katB* within food vacuoles.

Since we had observed a greater protection against ciprofloxacin than meropenem, we focused on investigating the underlying mechanism conferring protection towards ciprofloxacin during passage through ciliates. Because bacterivorous protists and macrophages challenge ingested bacteria with an oxidative burst in the phagosome [7], we first examined ROS production within the food vacuoles of *T. elliotti* by different approaches. First, we monitored ROS production in *T. elliotti* grazing on bacteria pre-stained with two different fluorogenic ROS probes: CellROX Orange (Fig. 3) or DCFH-DA (Fig. S5), which emit bright orange-red (570 nm) or green (532 nm) fluorescence, respectively, upon oxidation by ROS. Bacteria loaded with CellROX Orange exhibited a steady increase in 570-nm fluorescence when co-incubated with *T. elliotti* (Fig. 3 A), similar to that shown by bacteria exposed 0.02% H_2_O_2_, thus suggesting ROS production and subsequent oxidation of CellROX Orange. In contrast, no increase in fluorescence signal was detected in CellROX-stained bacteria incubated in the absence of ciliates. Treatment of ciliates with either VAS2870 (NOX inhibitor) or 1400 W (iNOS inhibitor) prior feeding significantly decreased the fluorescence signal produced by CellROX-stained bacteria, supporting that CellROX Orange was oxidized by protozoan-derived ROS. Additionally, ROS generation within *T. elliotti* phagosomes was corroborated by fluorescence microscopy by feeding GFP-expressing *B. cenocepacia* AM031 to CellROX Orange-stained *T. elliotti*. Co-localization analysis revealed that 55% of food vacuoles containing GFP-labeled bacteria exhibited bright orange-red fluorescence produced by oxidized CellROX (Fig. 3 B), confirming the exposure of *B. cenocepacia* to ROS within protozoan phagosomes. No red fluorescence was observed in CellROX-stained ciliates incubated in the absence of bacteria (Fig. S4). Similar results were obtained when these experiments were performed with the ROS probe DCFH-DA instead of CellROX Orange (Fig. S5 A and B). In line with these results, RT-qPCR detected overexpression of genes encoding putative NOX (*EI719255, EI719258, EI702462*) and iNOS (*EI705236*) homologs in *T. elliotti* at 1 h upon feeding them with *B. cenocepacia* (Fig. 3 C). Lastly, ROS production by ciliates was also confirmed by feeding them with the ROS-sensing *B. cenocepacia* strain AM051. This biosensor strain carries the reporter *egfp* gene under the control of the ROS-responsive promoter of catalase B (P*_katB_*) (Table S1). As expected, GFP fluorescence signal increased when AM051 was incubated in presence of *T. elliotti* (Fig. 2 F) or exposed to 0.02% H_2_O_2_ (Fig. S6 E). Besides, microscopy confirmed the presence of green fluorescent AM051 bacteria within food vacuoles (Fig. S6 C). Overall, the results from these experiments clearly indicate that *B. cenocepacia* is exposed to an oxidative burst within *T. elliotti* phagosomes.

Considering ROS production within protozoan food vacuoles, and the known association between ciprofloxacin tolerance and the SOS response in *E. coli* [49]*,* we hypothesized that oxidative radicals encountered in the protozoan phagosome may promote persister switch by activating the SOS response in *B. cenocepacia*. To investigate that, we first confirmed SOS upregulation within protozoan food vacuoles. We used RT-qPCR to quantify the expression levels of SOS (*recA, lexA*) and oxidative stress (*katB, sodC, oxyR*) related genes in *B. cenocepacia* cells collected from *T. elliotti* EFVs at 3 and 24 h post-feeding. Compared to bacteria incubated in the absence of ciliates, the relative expression of *recA*, *lexA*, *katB* and *sodC* was >2-fold higher in bacteria isolated from EFVs (Fig. 3 D). Therefore, upregulation of genes encoding catalase KatB and superoxide dismutase SodC, two enzymes involved in detoxification of H_2_O_2_ and O_2_^.-^, respectively, confirmed again that bacteria were exposed to ROS within *T. elliotti* food vacuoles. Likewise, overexpression of genes encoding the two SOS regulators *recA* and *lexA* suggested activation of the SOS response. Upon DNA damage, activated RecA protein triggers the SOS response by inducing self-cleavage of the autoregulated repressor LexA, thus releasing the transcription of LexA-regulated genes (*recA*, *lexA* and *uvrA*, among others), increasing their mRNA levels in the cell [50]. Supporting our data, similar results were obtained for *B. cepacia* cells recovered from *T. elliotti* EFVs (Fig. S7). Additionally, upregulation of *recA* in ingested bacteria was confirmed by feeding the reporter strain *B. cenocepacia* FA161 (which expresses *egfp* under the control of the *recA* promoter) to *T. elliotti*. A dramatic increase in GFP fluorescence was observed upon 4 h of co-incubation compared to the strain incubated in the absence of ciliates (Fig. 3 E). In addition, green fluorescent bacteria were observed inside intracellular food vacuoles and EFVs as well. Indeed, FA161 exhibited a brighter GFP fluorescence upon passage through *T. elliotti* when compared to bacteria incubated in the absence of ciliates (Fig. S6). Therefore, these results indicated that conditions encountered within the protozoan food vacuoles trigger the SOS response in *B. cenocepacia*.

**Figure 4:**
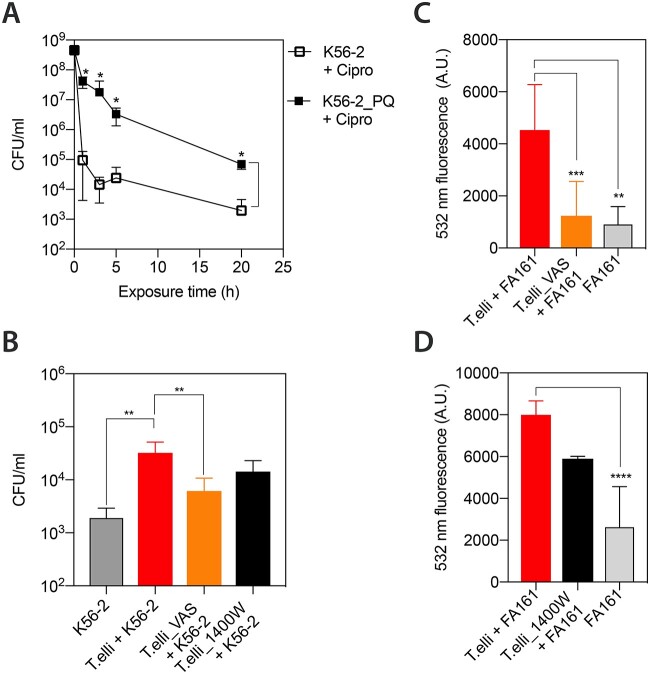
Protist-produced ROS induce persister switch in *B. cenocepacia*. (A) Time killing assay for log-phase K56–2 bacteria exposed to 40 μg/ml (10× MIC) ciprofloxacin in LB after being pre-treated with paraquat (PQ, filled square) or not (control, empty square) for 30 min before adding the antibiotic. Sublethal pre-challenge with PQ in LB enhanced antibiotic tolerance in planktonic *B. cenocepacia* cells. The antibiotic caused a bi-phasic killing curve indicating the presence of persisters tolerant to ciprofloxacin. (B) Formation of *B. cenocepacia* persisters within *T. elliotti* food vacuoles. Survival percentage of K56–2 collected from digestive vacuoles from ciliates that were non-treated (control) or pretreated for 1 h with 10 μM VAS2870 (NOX inhibitor) or 1400 W (iNOS inhibitor) prior feeding with bacteria. After 3 h of co-incubation, intracellular bacteria were released from food vacuoles with 1% Triton-X100 and exposed to ciprofloxacin (10× MIC) in LB for 3 h at 37°C. The percentage of bacteria that survived antibiotic (persisters) was estimated by CFU counts after plating on LB agar. VAS2870 and 1400 W decreased the number of intracellular persisters by >5-fold (*P* = .0011) and > 2-fold (*P* = .065), respectively, when compared to persisters recovered from untreated ciliates. (C-D) GFP signal of SOS-reporter strain FA161 (P*_recA_-egfp*) incubated with untreated ciliates (control) or those pretreated with VAS2870 or 1400 W prior feeding. After 3.5 h of co-incubation with bacteria, ciliates were harvested, lysed with 1% Triton-X100 and GFP fluorescence of released bacteria was measured. Bacteria collected from untreated ciliates exhibited 5-fold higher GFP fluorescence than those recovered from VAS2870-treated ciliates (*P* = .0002), confirming than NOX contributes to SOS activation within protozoan food vacuoles. Represented data correspond to the average of three independent experiments. Statistical significance (^*^P < .05, ^*^^*^P < .01, ^*^^*^^*^^*^P < .001) was determined by using the unpaired t Student’s test.

We tested whether ROS exposure promotes antibiotic tolerance in *B. cenocepacia*. Log-phase bacterial cultures were pre-treated with sub-lethal doses (1 mM) of the ROS-generating agent paraquat (PQ) for 30 min prior to exposure to high concentrations (10× MIC) of ciprofloxacin. Control experiments confirmed that PQ pretreatment did not cause bacterial death, as expected (Fig. S8 B). PQ is a redox-cycling compound that produces superoxide (O_2_^.-^) and subsequently H_2_O_2_, two of the ROS produced in the phagosome during the respiratory burst [36]. PQ pretreatment increased the number of bacteria (persisters) that survived 3 and 20 h exposure to ciprofloxacin by 3 and 2 log_10_, respectively (Fig. 4 A). Again, we observed the characteristic biphasic killing curve, suggesting that the surviving bacteria were indeed persisters. Then, given that a ROS challenge induced persister formation in LB medium, we investigated if blocking ROS production in the protozoan phagosome reduces the number of persisters recovered from *T. elliotti*. To assess that, we pretreated ciliates with either 10 μM VAS2870 (NOX inhibitor) or 100 μM 1400 W (iNOS inhibitor) for 1 h prior feeding them with *B. cenocepacia* K56–2. After 3 h, we lysed the ciliates and exposed the released bacteria to 40 μg/ml (10× MIC) ciprofloxacin for 3 h. Importantly, NOX suppression significantly (*P* = .0011) decreased by >5-fold the number of intracellular persisters recovered from food vacuoles, while inhibition of iNOS caused a > 2-fold reduction (*P* = .065) (Fig. 4 B). Hence, these results support that an oxidative burst induces persister within protozoan food vacuoles and suggest that *T. elliotti* NOX has a greater contribution compared to iNOS. In line with this, we confirmed that oxidative radicals generated by protozoan NOX and iNOS induce *B. cenocepacia* SOS response by feeding the SOS-reporter strain FA161 (which carries the reporter construct P*_recA_-egfp*) to ciliates pretreated with VAS2870 (NOX inhibitor) or 1400 W (iNOS inhibitor) for 1 h. Untreated ciliates served as control. After 3.5 h of co-incubation with bacteria, ciliates were lysed and GFP fluorescence of released bacteria was measured. Inhibition of protozoan NOX and iNOS reduced the expression of GFP by 5-fold *(P* = 00002) and 1.3-fold (*P* = .06), respectively, compared to bacteria collected from non-treated ciliates (Fig. 4 C and D). This result agrees with the fact that VAS2870 caused a greater reduction in oxidative radical production (Fig. 3 A) and persister formation than 1400 W (Fig. 4 B).

**Figure 5:**
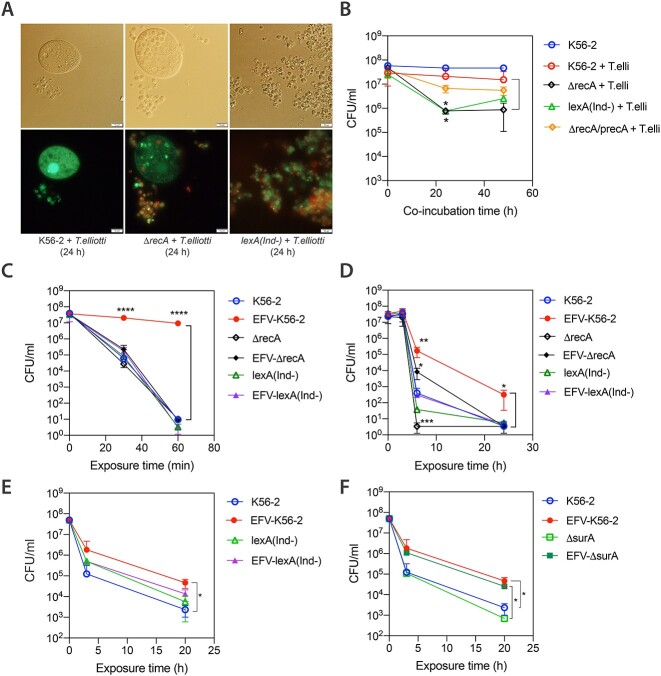
Impaired intracellular survival and stress resistance/antibiotic tolerance of WT and SOS-deficient mutant strains of *B. cenocepacia* K56–2 after passage through ciliates. (A) Fluorescence microscopy images of EFV-packaged bacteria stained with Syto9 and PI after 24 h of co-incubation with *T. elliotti*. EFVs produced by *T. elliotti* grazing on the SOS-deficient mutants Δ*recA* or *lexA(Ind-)* contained a higher proportion of membrane-damage bacteria (PI-positive, red fluorescence) than EFVs produced by ciliates grazing on wild-type K56–2 (EFVs filled with green bacteria, Syto9-positive, PI-negative). This result indicates that Δ*recA* or *lexA(Ind-)* mutations compromise *B. cenocepacia* ability to survive intracellular digestion within ciliates. (B) Bacterial survival under predation by *T. elliotti*. Ciliates were incubated with bacteria (ratio 1:200 protist:bacteria) in buffer. At indicated time points, ciliates were lysed with 1% Triton-X100 and surviving bacteria were enumerated by CFU counts on LB agar plates. Again, mutations in the SOS response regulators caused impaired intracellular survival (>97% bacteria were killed, ~2 log_10_ reduction of Δ*recA,* 1.7 log_10_ reduction of or *lexA(Ind-)* populations) within ciliates compared to the wild-type strain K56–2 (27% bacteria were killed, 0.1 log_10_ reduction). Complementation of the *recA* gene in Δ*recA* strain restored intracellular survival to wild-type levels. (C-F) Survival rate of bacteria released from EFVs (filled symbols) and control planktonic bacteria (empty symbols) subjected to 0.02% H_2_O_2_ (C), desiccation (D), or 80 μg/ml (20× MIC) ciprofloxacin (E-F). At indicated time points, bacteria were plated on LB agar and survivors were quantified by CFU counts. Mutations in the SOS regulators decreased resistance to H_2_O_2_ and desiccation, as well as the percentage of persisters tolerant to ciprofloxacin formed within protozoan food vacuoles compared to wild-type strain (E). In contrast, the mutant Δ*surA* exhibited a tolerance to ciprofloxacin similar to that of the wild-type strain K56–2 after passage through ciliates (similar number of persisters) (F). Data represent the average of at least three independent biological replicates. Statistical significance (^*^P < .05, ^*^^*^P < .01, ^*^^*^^*^^*^P < .001) was determined with the unpaired t Student’s test.

### The SOS response is involved in *B. cenocepacia* intracellular survival and enhanced antibiotic/stress tolerance of expelled bacteria

To confirm the role of the SOS response in persister formation within protozoan food vacuoles we constructed the two isogenic SOS-deficient mutant strains Δ*recA* and *lexA(Ind-)*. We fed them to ciliates and monitored their intracellular survival and enhanced stress resistance/antibiotic tolerance after passage through ciliates. RecA (inducer) and LexA (repressor) are the master regulators of the SOS response [51]. Strain Δ*recA* expresses a non-functional truncated RecA protein, whereas strain *lexA(Ind-)* carries a LexA variant resistant to RecA cleavage, thus preventing induction of SOS response while maintaining RecA activity. The SOS-deficient phenotype of each strain was confirmed by assessing their higher susceptibility to UV exposure compared to the parental wild type strain K56–2 (Fig. S9 B). Additionally, RT-qPCR assays corroborated the lack of SOS upregulation when both mutant strains were exposed to mitomycin C, a well-known inducer of the SOS response (Fig. S9 C). *B. cenocepacia* intracellular survival was significantly impaired by mutations blocking the bacterial SOS response (Fig. 5 A and B), suggesting that bacteria indeed face DNA damage within protozoan food vacuoles. *T. elliotti* killed >97% (~2 log_10_ reduction) of the Δ*recA* and *lexA(Ind-)* populations, while it digested 27% (~0.1 log_10_ reduction) of the wild type population (Fig. 5 B). The higher survival rate of strain *lexA(Ind-)* (3%) compared to Δ*recA* (1.3%) suggests that RecA-mediated homologous recombination likely contributes to DNA repair in *B. cenocepacia* during passage through ciliates. Live/Dead BacLight staining corroborated impaired survival for SOS-deficient mutants, as the EFVs produced by ciliates grazing on either Δ*recA* or *lexA(Ind-)* were filled with many membrane-damaged bacteria (PI-positive cells), whereas EFVs produced by ciliates grazing on the WT K56–2 strain contained mostly (>95%) membrane intact cells (Syto9-positive and PI-negative cells, Fig. 5 A). Furthermore, EFV-released SOS-mutants that survived intracellular digestion showed similar resistance to H_2_O_2_ and desiccation as the parental strain K56–2 incubated in Tris–HCl buffer (Fig. 5C and D). Likewise, the number of persisters tolerant to ciprofloxacin did not increase during passage through ciliates when the *lexA(Ind-)* strain was used (Fig. 5 E), as the number of *lexA(Ind-)* persisters wihin EFVs was similar to that of the *lexA(Ind-)* strain incubated in buffer. Because the Δ*recA* mutant exhibited a stronger defect in intracellular survival compared to *lexA(Ind-),* we only evaluated persister formation during passage through ciliates with the *lexA(Ind-)* strain. Overall, these results indicate that SOS activation contributes to stress adaptation and persister formation within protozoan food vacuoles. Supporting these results, a Δ*surA* mutant strain with has higher susceptibility to ciprofloxacin (MIC = 2 μg/ml) than K56–2 (MIC = 4 μg/ml) and similar to *lexA(Ind-)* (MIC = 2 μg/mL) but it retains an intact SOS response (Fig. S10) increased its tolerance to ciprofloxacin after passage through ciliates (Fig. 5 F). Therefore, the absence of a functional SOS response is likely the reason that *lexA(Ind-)* mutant did not increase its tolerance to this antibiotic after passage through ciliates.

## Discussion

It has been proposed that the induction of stress responses within protozoan food vacuoles helps bacteria to adapt themselves to the stress conditions that they will encounter in the human host [52]. Our study evidences that *B. cenocepacia* survives phagocytosis by ciliates found in the natural and built environments, and viable bacteria are expelled packaged in fecal pellets (or EFVs) with enhanced tolerance towards harsh conditions such as desiccation, oxidative stress, and antibiotics, which may significantly contribute to the persistence and transmission of this opportunistic pathogen. We show that genes encoding antioxidant enzymes are upregulated during passage through ciliates. This increases bacterial resistance to ROS, helping them to survive desiccation, oxidizing sanitizers, or host defenses. Whereas previous studies demonstrated that packaging of bacteria in EFVs protects them from stress and antibiotics [15–17], we have identified that oxidative burst in the protozoan phagosome promotes stress resistance and persister switch by triggering the SOS response in *B. cenocepacia*.

In the last decade, different mechanisms contributing to persister formation have been identified. These include activation of toxin–antitoxin (TA) systems, the stringent response and (p)ppGpp signaling, RpoS-mediated general stress response and the SOS response [46, 53–55] . While *rpoS* exhibited similar expression levels in K56–2 collected from EFVs or incubated in Tris–HCl buffer, *recA* and *lexA* were significantly upregulated, suggesting activation of the SOS response during passage through ciliates. Accordingly, the P*_recA_-egfp* transcriptional fusion was strongly induced in *B. cenocepacia* encased within *T. elliotti* food vacuoles. In line with this, an RNA-seq study carried out in our lab did not detect upregulation of *rpoS, relA* and genes encoding known TA systems in K56–2 bacteria collected from *T. elliotti* EFVs [56]. Therefore, our data suggest that RpoS-stress response, (p)ppGpp signaling and TA systems may not be involved in formation of persisters tolerant to ciprofloxacin within ciliate food vacuoles, while the SOS response appears to play a crucial role. Previous studies have shown that the phagosomal environment of amoebae and macrophages favors persister switch in *L. pneumophila* in a process controlled by the Legionella quorum system (Lqs) [34]. Likewise, macrophages have been found to induce antibiotic tolerance of internalized *Salmonella* serovar *typhimurium*, *Mycobacterium tuberculosis*, and *Staphylococcus aureus* [36, 57, 58], suggesting that persister switch may be a widespread phenomenon in intracellular bacteria surviving digestion within macrophages. Oxidative killing in the phagosome relies on generation of ROS and RNS primarily through the phagosomal NOX complex and the cytosolic iNOS [5]. Our data revealed that genes encoding NOX and iNOS homologs in *T. elliotti* are quickly upregulated upon phagocytosis of bacteria. Fluorogenic probes detected production of oxidative species within food vacuoles filled with bacteria. Inhibitors of protozoan NOX and iNOS reduced the fluorescence signal, supporting their role in ROS/RNS production. Inhibition of NOX and iNOS also decreased the number of intracellular persisters tolerant to ciprofloxacin, indicating that protist-derived ROS/RNS are likely involved in persister formation within food vacuoles. NOX seems to have a major contribution, since its inhibition caused a greater reduction in ROS generation and persister formation. This is consistent with previous studies showing that host defense against *B. cepacia* primarily relies on NOX rather than iNOS in the mouse model [59].

Phagosomal ROS generated by macrophages induce antibiotic tolerance in intracellular *S. aureus* [36]. Oxidative radicals may inhibit bacterial growth, which has been associated with antibiotic tolerance [60]. However, recent studies have shown that the induction of antibiotic tolerance cannot be solely attributed to growth inhibition by ROS [36]. Besides, we believe that growth arrest alone was not sufficient to induce ciprofloxacin tolerance in ingested K56–2 since starving bacteria incubated in Tris–HCl buffer for 24 h were rapidly killed by this antibiotic. Although persisters were initially defined as dormant cells in which essential targets that antibiotic corrupt are not active, recent studies have called this into question [31, 43], particularly within intracellular niches where bacteria must maintain their metabolic activity to withstand stressors imposed by the eukaryotic host cell [61, 62]. Moreover, persistence cannot be solely explained by growth arrest and reduced target activity for quinolones because they target topoisomerases and non-growing cells still possess transcriptional activity [46, 63]. Therefore, since both inhibition of *T. elliotti* NOX complex and mutations that abolished the SOS response significantly decreased the number of *B. cenoceapcia* persisters within EFVs, we propose that the SOS response triggered by oxidative burst leads to persister formation within *T. elliotti* food vacuoles. The fact that we observed lower upregulation of P*_recA_-egfp* in ingested bacteria when ciliates were pretreated with the NOX inhibitor supports SOS activation by phagosomal ROS. Besides, this finding is consistent with studies from other laboratories showing that activation of the SOS response confers tolerance to fluoroquinolones and beta-lactams (ampicillin) in *E. coli* and *S. aureus* [49, 64, 65]. Beyond a DNA repair process, the SOS response is now considered as a powerful strategy that allow bacteria to better adapt and survive to changing environments, regulating genes involved in mutagenesis, virulence, antibiotic resistance dissemination, horizontal gene transfer, and inter-species competition [50, 66]. Our work demonstrates that this pathway contributes to *B. cenocepacia* intracellular survival within ciliates and the subsequent enhanced stress resistance and antibiotic tolerance of egested bacteria. Importantly, because the SOS response is a conserved mechanism, our study suggests that protozoan food vacuoles likely constitute an important reservoir that promotes the switch to persister in bacteria that survive intracellular digestion. Furthermore, it has been recently shown that oxidative radicals produced within protozoan food vacuoles facilitate genetic diversification in *V. cholerae* through SOS-induced DNA integration [67]. Exchange of antibiotic resistance genes has been shown to be favored in protozoan food vacuoles, where different bacterial species accumulate under conditions that favor major mechanisms for horizontal gene transfer [67–72]. Therefore, over the last years evidence is accumulating about the role of protozoan food vacuoles as privileged niches that foster genetic diversification and adaptation in bacteria. Deciphering the molecular mechanisms governing this phenomenon is crucial for the development of novel strategies to mitigate pathogen dissemination by modulating bacteria–protist interactions.

## Supplementary Material

Moron2023_Supp_Material_REVISED

## Data Availability

Data sets have been deposited at https://figshare.com/projects/Moronetal2024/193346 Any further information or additional data will be made available upon request.
