# Supplementary material for: Protozoan predation enhances stress resistance and antibiotic tolerance in Burkholderia cenocepacia by triggering the SOS response
Source: ISME J. 2024 Jan 28;18(1):wrae014. doi: 10.1093/ismejo/wrae014 (PMC10944698; doi:10.1093/ismejo/wrae014)
Supplement: Moron2023_Supp_Material_REVISED [file moron2023_supp_material_revised.pdf]

Supplementary Material

Protozoan predation enhances stress resistance and antibiotic tolerance in *Burkholderia cenocepacia* by triggering the SOS response

Álvaro Morón, Alaa E. Tarhouchi, Iván Belinchón, Juan M. Valenzuela, Patricia de Francisco, Ana Martín-González and Francisco Amaro*

Department of Genetics, Physiology and Microbiology. Faculty of Biological Sciences, Complutense University of Madrid. 28040 Madrid. Spain.

*To whom correspondence should be addressed famaroto@ucm.es

Supplementary Methods

Construction of reporter and mutant strains

Unmarked non-polar deletion mutants were constructed by using the approach of Flannagan and co-workers [1], which is based on the homing nuclease I-SceI. To delete the *recA* gene, the knockout plasmid pFA094 was generated by PCR amplification with Phusion HF DNA polymerase (Thermo Scientific) of the upstream and downstream flanking regions of *recA* from *B. cenocepacia* K56-2 gDNA (locus tag K562_10985, accession number NZ_CP053300) and primers FA198Fw to FA200Rv, incorporating the XbaI and EcoRV restriction sites to insert them into the pGPI-SceI plasmid. Plasmid pFA094 was transferred into *B. cenocepacia* K56-2 by tri-parental mating using *E. coli* HB101 harbouring plasmid pRK2013 as helper strain. Briefly, overnight cultures of donor, helper and recipient strains were resuspended in fresh SOB medium and combined in a 3:3:1 ratio, respectively. Mating mixtures were spotted onto a nitrocellulose filter overlaid on LB agar plates and incubated at 37 ºC for 16 h. Exconjugants were selected on LB medium with 100 μg/ml trimethoprim and 50 μg/ml gentamicin and screened by colony PCR. Positive clones were conjugated with *E. coli* carrying plasmid pDAI-SceI-SacB to induce a second crossover event. Tetracycline-resistant trimethoprim-sensitive exconjugants were selected and confirmed for *recA* deletion by PCR analysis. The deletion mutants were then cured from pDAI-SceI-SacB by growing in LB overnight and screening for loss of tetracycline resistance. The Δ*recA* phenotype was confirmed by its higher susceptibility to UV compared to the wild type strain K56-2. The *B.*

cenocepacia Δ*recA* mutant was complemented as follows. The coding region of *recA* was PCR amplified with primers FA219Fw and FA220Rv from gDNA of K56-2 and cloned into NdeI/XbaI sites of plasmid pSCrhaB2plus [2]. The resulting plasmid, named pFA107, was introduced into K56-2 Δ*recA* strain by tri-parental mating as described above. Transconjugants harbouring the complementing plasmid were selected on trimethoprim and gentamicin containing plates and confirmed by PCR. This strain was named as FA108.

The *lexA(Ind-)* mutant strain was obtained by gene replacement. To generate the *lexA*(S119A) mutation cassette, two fragments containing the 500 bp upstream region with the complete *lexA* CDS (locus tag K562_12117, accession number NZ_CP053300) and the 500 bp downstream region were PCR amplified with Phusion HF DNA polymerase (Thermo Scientific) from K56-2 gDNA using primers FA225Fw-FA226Rv and FA227Fw-FA228Rv, respectively. The tetracycline cassette was PCR amplified from pDAI-SceI-SacB using primers FA223Fw and FA224Rv. Then, the three fragments were PCR assembled together using the 20 bp homology regions in primers FA226Fw and FA227Rv, and introduced into pGPI-SceI by restriction cloning with XbaI and EcoRI (Thermo Fisher Scientific). The point mutation *lexA*(S119A) was introduced using the Q5-Site-directed mutagenesis kit (New England Biolabs) with primers FA229Fw and FA230Rv. The resulting mutant allele was verified by Sanger sequencing with primers FA236Fw and FA237Rv. Plasmid carrying the *lexA*(S119A)::Tet^r cassette was named pFA118 and was delivered to K56-2 by triparental mating as described before. Tetracycline-resistant and trimethoprim sensitive exconjugants were selected and the presence of the mutant allele *lexA*(S119A) was confirmed by PCR and DNA sequencing. The *lexA(Ind-)* phenotype was confirmed by its higher susceptibility to UV compared to the wild type strain K56-2.

The FA115 and AM031 strains were generated by introducing pFA158 and pAM028 into K56-2 by triparental mating as described before and selecting for trimethoprim-gentamicin and trimethoprim-chloramphenicol resistant exconjugants, respectively. pFA158 was generated by cloning the *dsRed2* coding sequence into NdeI/XbaI sites of plasmid pSCrhaB2plus. *dsRed2* coding sequence was PCR amplified from plasmid pUC18T-mini-Tn7T-Tp-dsRedExpress [3] with primers FA265Fw and FA266Rv. pAM028 was constructed by replacing the I-*SceI* gene in pAH-25 [4] by *egfp*, which was previously PCR amplified with primers FA218Fw and FA219Rv and digested with NdeI and XbaI.

The FA161 (P_{recA}-*egfp)* and AM051 (P_{katB}-*egfp)* reporter strains were generated by introducing pFA160 and pAM050, respectively, into K56-2 by triparental mating as described above. pFA160 and pAM050 were generated by replacing the P_{S7} promoter in pMLS7eGFP [5] with the promoter of *recA* or *katB*, respectively. The promoter regions were previously

PCR amplified using primers FA289Fw/FA290Rv and FA471Fw/FA472Rv from K56-2 gDNA with Phusion HF DNA polymerase (Thermo Scientific).

The oligonucleotides used in this study are listed in Table S3.

Determination of MIC

The MIC was measured by performing the microdilution test and following CLSI reference methods [6]. Bacterial growth was estimated by measuring OD_{600} at 24 h with a TECAN Infinite MPlex plate. We determined that a given antibiotic concentration prevented bacterial growth when it reduced bacterial growth to an OD_{600} comparable to that of the negative control well (LB medium with no bacteria) after 20 h of incubation in static conditions.

UV stress assays

Log-phase cultures (OD_{600}=0.3) of wild type and mutant strains were washed and suspended in fresh LB medium. Serial 10-fold dilutions were prepared in fresh LB medium and spotted on the surface of LB agar plates. The plates were then irradiated with 222 nm UVC light at a dose of 100 W/cm^2 for different times using a UVO-Ccrosslinker (EQUILAB, SL, Madrid). After irradiation, plates were incubated at 37 ºC for 24 h. The survival percentage of the population was calculated as the number of CFUs divided by the total number of CFUs in the non-irradiated population.

Supplementary Tables

Table S1. Bacterial strains and protists used in this study

Strain	Description	Source
Bacteria		
E. coli		
HB101/pRK2013	HB101 strain harbouring pRK2013	M. J. Grillo (IDAB-CSIC)
BW25141	F-, *Δ(araD-araB)567*, *ΔlacZ4787*(*::rrnB*-3), *Δ(phoB-phoR)580*, λ^-, *galU95*, *ΔuidA3::pir*$^+$, *recA1*, *endA9*(del-ins)*::FRT*, *rph-1*, *Δ(rhaD-rhaB)568*, *hsdR514*	A. Sánchez-Gorostiaga (iMiDRA, Spain)
B. cenocepacia		
K56-2	Clinical isolate from a CF patient, ET12 lineage	BCCM/LMG Bacterial Collection
Δ*recA*	Δ*recA* derivative of K56-2	This study
FA108	Δ*recA* harbouring pFA107 plasmid expressing WT *recA* allele under the control of P_{rhaBAD} promoter	This study
lexA(*Ind-)*	*lexA*(Ind-) derivative of K56-2	This study
FA115	K56-2 expressing *dsRed* under the control of P_{rhaBAD} promoter	This study
FA161	K56-2 harbouring pFA160; expressing *egfp* under the control of P_{recA} promoter	This study
AM031	K56-2 harbouring pAM028; expressing *egfp* under the control of constitutive *P1* promoter derived from *dhfrIIb*	This study
AM051	K56-2 harbouring pAM050; expressing *egfp* under the control of P_{katB} promoter	This study
Δ*surA*	Δ*surA* derivative of K56-2	[7]
B. cepacia		
ATCC25416	Environmental isolate (source *Allium cepa*)	Spanish Type Culture Collection (CECT)
Protists		
Tetrahymena elliotti 4EA	Ciliate, laboratory strain	Tetrahymena Stock Center Source: environment
Tetrahymena sp T2305B2	Ciliate, wild isolate	Hospital sink drain (F. Amaro, unpublished)
Colpoda sp CE36	Ciliate, wild isolate	Domestic sink drain (F. Amaro, unpublished)

Table S2. Plasmids used in this study

Plasmid	Description	Reference
pRK2013	ori$_{colE1}$, RK2 derivative, Kan^{r} mob^{+} tra^{+}	[8]
pGPI-SceI	ori$_{R6K}$, mob^{+},Tp^{r}, I-SceI-I restriction site	[1]
pDAI-SceI-SacB	ori$_{pBBR1}$, mob^{+},Tet^{r}, expressing I-Sce-I and sacB	[1]
pSCrhaB2plus	pSCrhaB2 with rhaI1-rhaI1 permutation of rhaS binding sites upstream of P_{rhaBAD} and bacteriophage T7 gene 10 stem loop inserted upstream of native rhaBAD 5′ UTR	[2]
pFA094	pGPI-SceI with regions flanking K56-2 *recA*	This study
pFA107	pSCrhaB2plus with K56-2 *recA* gene cloned	This study
pFA118	pGPI-SceI with *lexA*(S119)::Tet^{r}	This study
pFA158	Derived from pSCrhaB2plus with *dsRed2* cloned	This study
pAM028	Derived from pAH25-SceI; I-*SceI* gene replaced by *egfp*, Cm^{r}	This study
pFA160	Derived from pMLS7eGFP; P_{S7} promoter replaced by P_{recA} promoter from K56-2, Tp^{r}	This study
pAM050	Derived from pMLS7eGFP; P_{S7} promoter replaced by P_{katB} promoter from K56-2, Tp^{r}	This study

Table S3. Oligonucleotides used in this study. Oligonucleotides were synthesized by Integrated DNA Technologies.

Primer name	Sequence (5' → 3')	Application
FA198Fw	TACGTCTAGAAGGATCAGCAGGTCGAAAGT	Generation of plasmids and mutant strains
FA199Rv	TTTTCTCGAGAGCGTGAGCGTGGTCTTA	
FA194Fw	TTTTCTCGAGTGTACGGCGAAGGCATTT	
FA200Rv	TTTTGATATCGCTGCGCGTATAGACATCTT	
FA218Fw	ATTAGACCATATGGTGAGCAAGGGCGAGGA	
FA219Rv	CGTCTAGATTACTTGTACAGCTCGTCCATG	
FA219Fw	ATTAGACCATATGACCGCCGAGAAGAGCAAG	
FA220Rv	CG TCTAGA TCACTCTTCTTCGTCCATCG	
FA223Fw	GGCTCTGCTGTAGTGAGTGG	
FA224Rv	GCGAAGAAGTTGTCCATATTG	
FA225Fw	TCGCATCTAGACCGTGCGACTGCTTGAAAT	
FA226Rv	GCAACCCACTCACTACAGCAGAGCCTCAGAGTTCGCCCGAGC	
FA227Fw	TGGCCAATATGGACAACTTCTTCGCGTCTCGCTCAGGAGAACAT	
FA228Rv	TTTTGAATTCGACGCGTACGCATAGCC	
FA229Fw	GCGCGGCCTGGCGATGCGCGA	
FA230Rv	ACCTTCAGCAGGTAGTCGGGCTTGCTGG	
FA236Fw	GCGTCGAACCTCAAGTAACA	
FA237Rv	ACGTTGAAGGACCGAGAAAG	
FA265Fw	TTTTCATATGGCCTCCTCCGAGAACGTC	
FA266Rv	TCGATCTAGACTACAGGAACAGGTGGTG	
FA289Fw	TTTATCGATCGAACGGCTTGGCCATGTA	
FA290Rv	TTTCCATGGCCCGGAGCCCTTCTTGCTAT	
FA471Fw	TTTATCGATCATGGATCGGCGTCGTAG	
FA472Rv	TTTCCATGGGCTTTTTCTCCTTTACTTGCTCG	
gyrAFw	CGACGCAGATGAAGGAAGA	Quantitative RT-PCR
gyrARv	CATAGACCTTGACCCAGTACAC	
lexAFw	GAAGGACGGCCAGATCAT	
lexARv	TTTCGTAATCCGGGTTCTCC	
recAFw	TGTACGGCGAAGGCATTT	
recARv	CGATCTTCTCGCCGTTGTAG	
rpoSFw	CGTGATCCGCGAACTGAA	
rpoSRv	GGTCTTGCCGGTGAGATAG	
oxyRFw	TCGTCGCTCGAAACCATTC	
oxyRRv	GCACGTACGACAGCAGTT	
katBFw	AGTGGAGCAACGACTTCTTC	
katBRv	AATCACCTCGTCCGCATC	
sodCFw	TTCCTCGTCGCACGAAA	
sodCRv	CGACCAGGTTGTAGGTGAC	
TeACT1Fw	GTACCACAATGTTCCCTGGTAT	
TeACT1Rv	AGGAGGAGCAACAACCTTAATC	
EI719254Fw	AGGATATGCTGATGGTCCTTATG	
EI719254Rv	GGAGTAACACCTATTCCACCAC	
EI719255Fw	CGGTCCTTAATCGCTCATCTAAA	
EI719255Rv	GTGTATATCAAACCTGACTCCATCT	
EI719258Fw	CTAAAGAGAGCGGCGAAGAT	
EI719258Rv	GACTCCTTGCTTTGACCATTTC	
EI702462Fw	TATGATATGTGGTCCCTCTCCA	
EI702462Rv	CCAAACACTTCTTCGTGAACATC	
iNOSFw	GATCAAGGTAGCAAGGTCTATGT	
iNOSRv	ACACTTTCACCCATAGCAGTAG	

Supplementary Figures

Figure S1. Representative microscopy images of EFVs purified from *B. cenocepacia* K56-2 and *T. elliotti* co-cultures. **(A)** Purified EFVs before washing steps. **(B)** Aggregate of purified EFVs after washing and centrifugation steps as described in the Methods section.

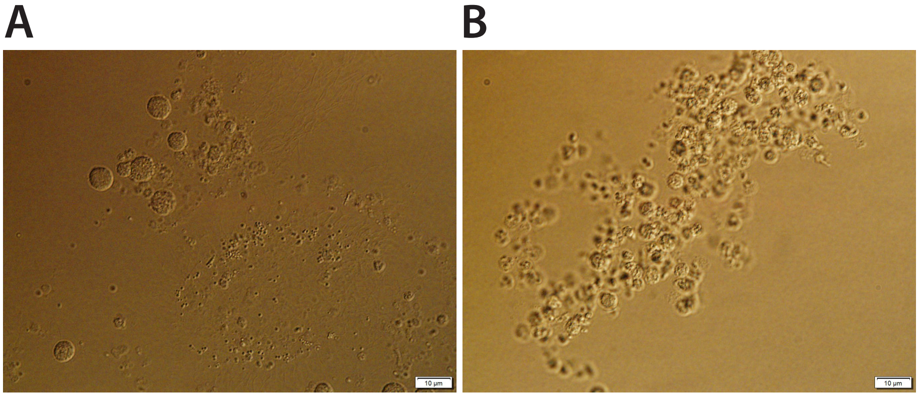

Figure S2. TEM micrographs of wild *Tetrahymena sp* T2305B2 grazing on *B. cenocepacia* K56-2. **(A)** Ciliate expelling EFVs (black arrows) laden with bacteria. **(B)** Aggregate of several EFVs (white arrows) and free bacteria.

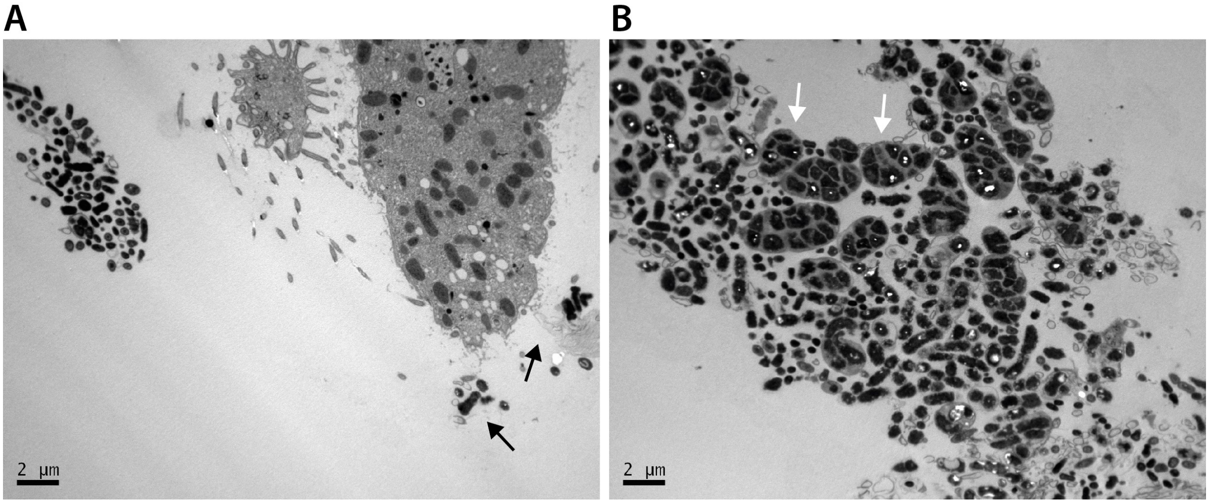

Figure S3. Biphasic killing curve obtained in follow-up persister assays. Bacteria that survived 20 h exposure to 40 µg/ml ciprofloxacin in persister assays were grown in fresh LB medium overnight, harvested and inoculated in LB supplemented with 40 µg/ml ciprofloxacin for 20 h for a follow-up persister assay. The number of surviving bacteria was estimated by CFU counting on LB plates. Data shown correspond to the avearge of two independent experiments. En each experiment three independent cultures were grown and exposed to the antibiotic.

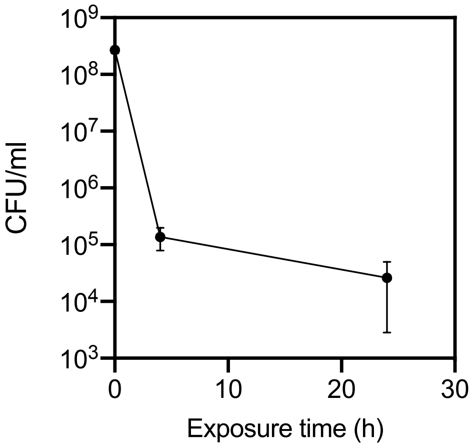

Figure S4. Representative fluorescence images of *T. elliotti* stained with CellROX Orange and incubated with *B. cenocepacia* AM031 **(A)**, 0.01 % H_2O_2 **(B)** or Tris-HCl buffer **(C)**. Red fluorescence produced by oxidized CellROX Orange was observed within H_2O_2-treated ciliates (B, positive control) and within protozoan food vacuoles containing GFP-expressing AM031 (A), supporting ROS production. Ciliates incubated in Tris-HCl buffer did not exhibit any green or red fluorescence (C, negative control).

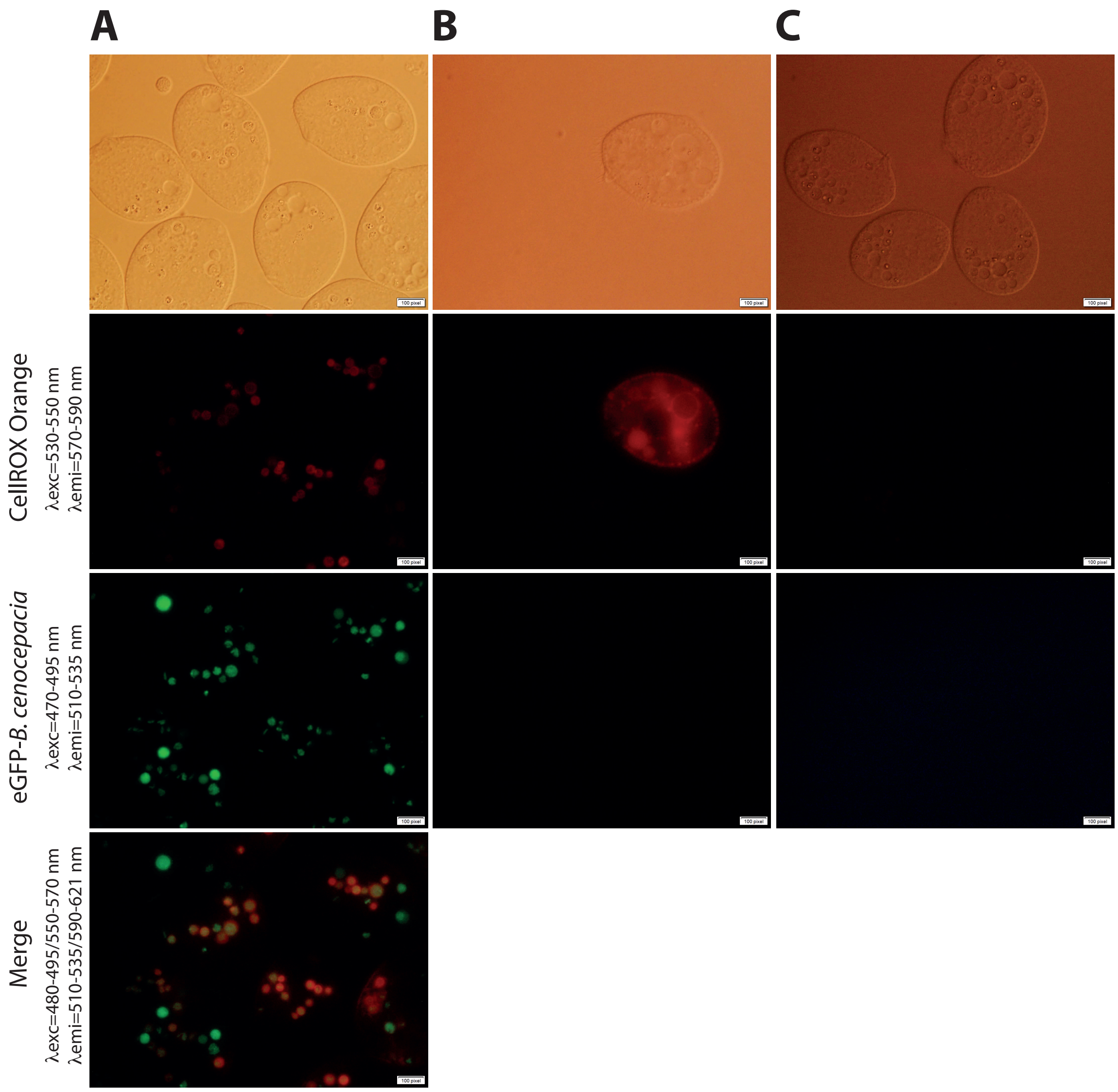

Figure S5. Detection of ROS production within protozoan phagosomes with the fluorogenic probe DCFH-DA. **(A)** Monitorization of ROS production as green (532 nm) fluorescence emitted by DCF-stained *B. cenocepacia* K56-2 ingested by *T. elliotti*. Bacteria were stained with DCFH-DA for 30 min, washed and fed to *T. elliotti*. A dramatic increase in the production of green fluorescence was detected for DCF-stained K56-2 cells that were incubated in the presence of *T. elliotti* (purple line) or 0.02% H_2O_2 (black line, positive control). **(B)** Co-localization of ROS and bacteria in protozoan food vacuoles was demonstrated by feeding dsRed-expressing K56-2 bacteria to DCF-stained *T. elliotti* (after 3 h co-incubation). Scale bar represents 10 µm. **(C)** Quantification of ROS production in *T. elliotti* cells pretreated with VAS2870 (NOX inhibitor) or 1400W (iNOS) prior feeding with DCF-stained bacteria. NOX inhibition led to lower increase in fluorescence signal, suggesting lower ROS production in VAS2870-treated ciliates (grey bar) compared to non-treated ciliates (purple bar).

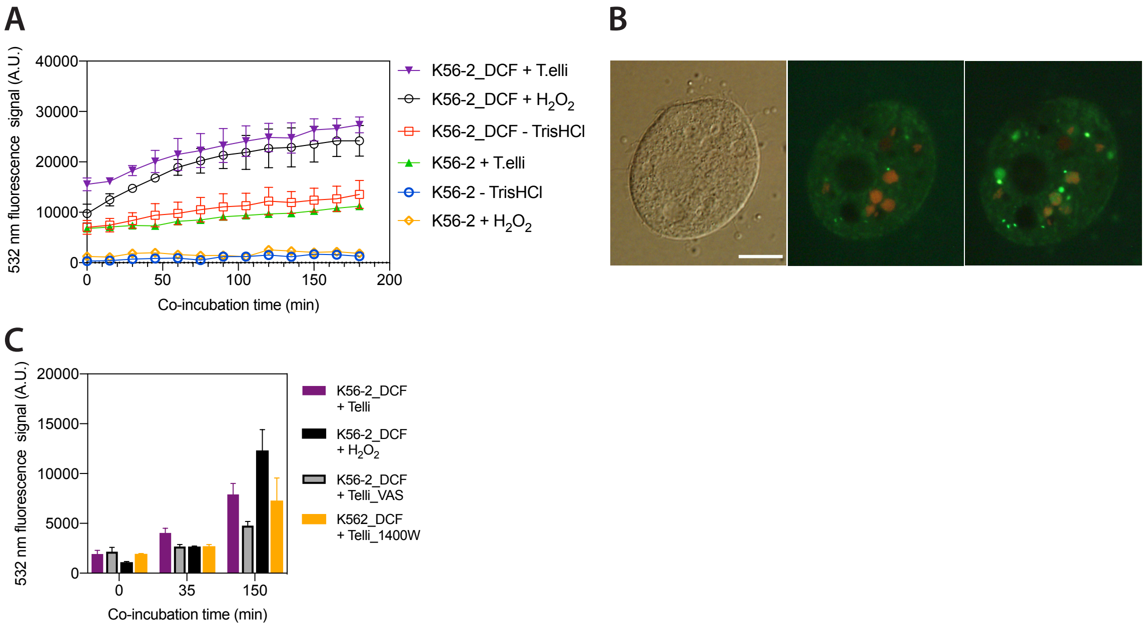

Figure S6. Representative fluorescence images of *B. cenocepacia* strains FA161 (A-B) and AM051 (C-E) incubated with (A, C) or without ciliates (B, D, E) for 4 h. Bacteria exhibited bright green fluorescence when encased within protozoan food vacuoles or EFVs, indicating upregulation of the P_{recA} (A) and P_{katB} (C) promoters. Bacteria incubated in the absence of ciliates displayed lower green fluorescence signal (B, D). Expression of the reporter construct P_{katB}-*egfp* was also induced by treating bacteria with H_2O_2 (E, positive control). Scale bar represents 10 μm.

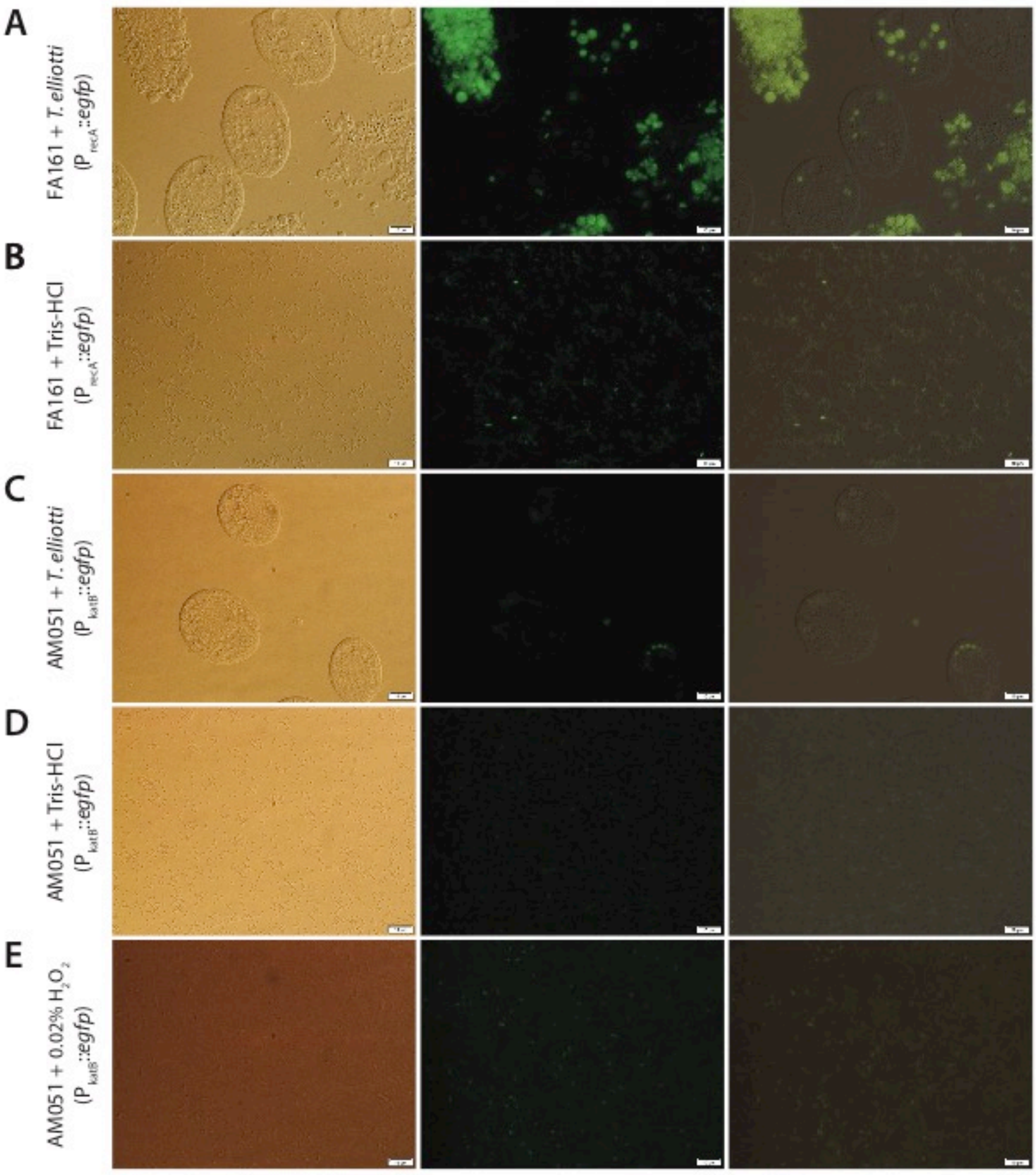

Figure S7. Upregulation of SOS response genes in *B. cepacia* ATCC25416 recovered from *T. elliotti* EFVs (24 h post-feeding). Ciliates were incubated with bacteria (ratio 1:200 protist:bacteria) in buffer. At 0 and 24 h ciliates and EFVs were lysed with 1% Triton-X100 and RNA was isolated from collected bacteria. Bacteria incubated in Tris-HCl for 24 h in the absence of ciliates served as control. The stars indicate significant differences (** $p < 0.01$) when compared to the control group (bacteria incubated in buffer for 24 h) according the unpaired t student's test.

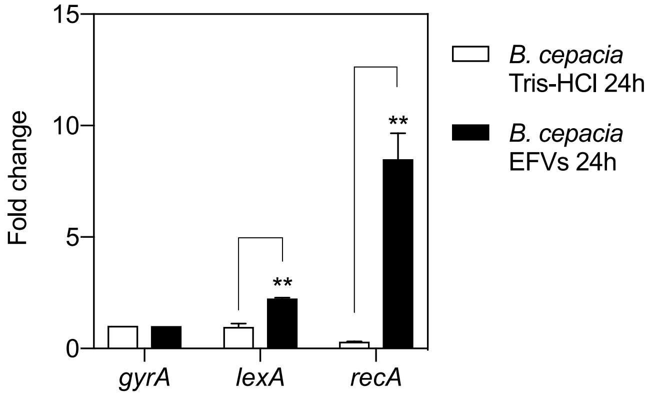

Figure S8. Paraquat (PQ) treatment in LB. **(A)** MIC assay for *B. cenocepacia* K56-2 exposed to different concentrations of PQ in LB for 24 h. Bacterial growth was estimated by measuring OD_{600} after 24 h in static conditions in a 96 well plate. **(B)** As expected, exposure to 1 mM PQ in LB for 30 min did not result in bacterial death. The number of surviving K56-2 bacteria was quantified by CFU counts on LB agar plates at 0 and 30 min after exposure to PQ.

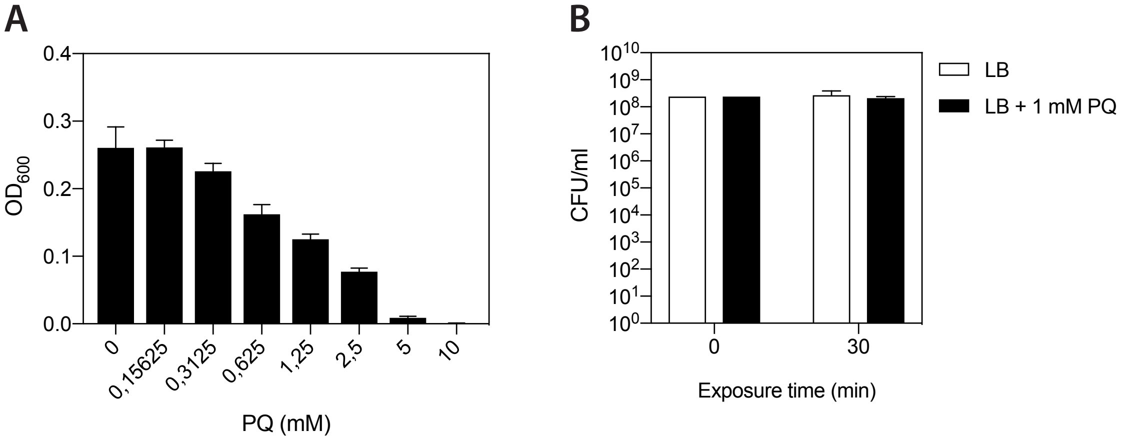

Figure S9. Corroboration of the SOS-deficient phenotype of mutant strains Δ*recA* and *lexA(Ind-)*. **(A)** Growth curves of strains K56-2, Δ*recA* and *lexA(Ind-)* in LB medium. **(B)** Survival assays of bacteria exposed to UVC (100 W/cm^2). SOS-deficient mutants exhibited higher susceptibility to UV compared to parental strain K56-2. Complementation of the *recA* gene in Δ*recA* strain restored UV resistance to wild-type levels. **(C)** Expression levels of *recA* and *lexA* genes in bacteria treated with 2 μg/mL mitomycin C (MMC). MMC failed to induce the SOS response (upregulation of *recA* and *lexA* genes) in strains Δ*recA* and *lexA(Ind-)*. Data represent the average of at least three independent biological replicates. Significant differences (* p < 0.05, ** p < 0.01, *** p < 0.005) were calculated using one-way ANOVA and Dunnett's multiple comparison test.

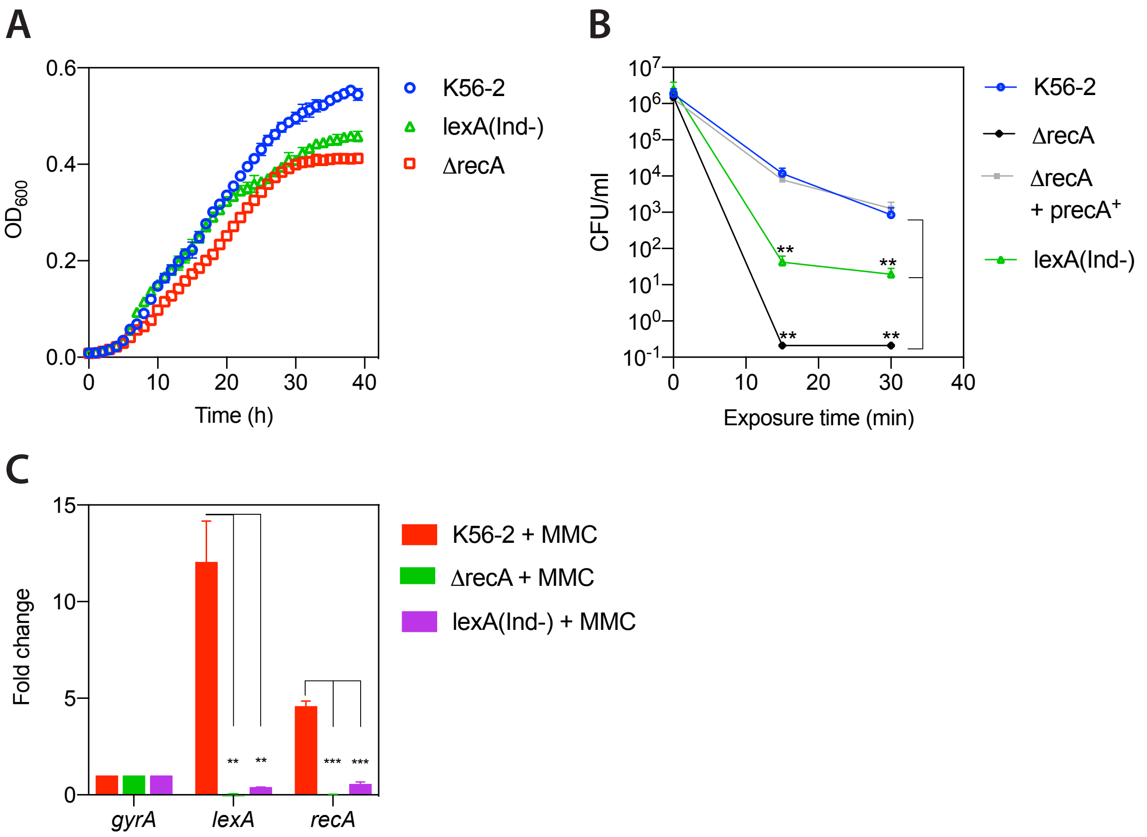

Figure S10. Ciprofloxacin minimal inhibitory concentration (MIC) assay of SOS-deficient mutants (Δ*recA* and *lexAInd-*), Δ*surA* and parental strain K56-2. MIC was measured by performing the microdilution test and following CLSI reference methods [6]. Bacterial growth was estimated by measuring OD_{600} at 24 h. MIC was determined as 4 µg/mL for K56-2, 2 µg/mL for *lexA(Ind-)*, <2 µg/mL for Δ*surA* and 1 µg/mL for Δ*recA.*

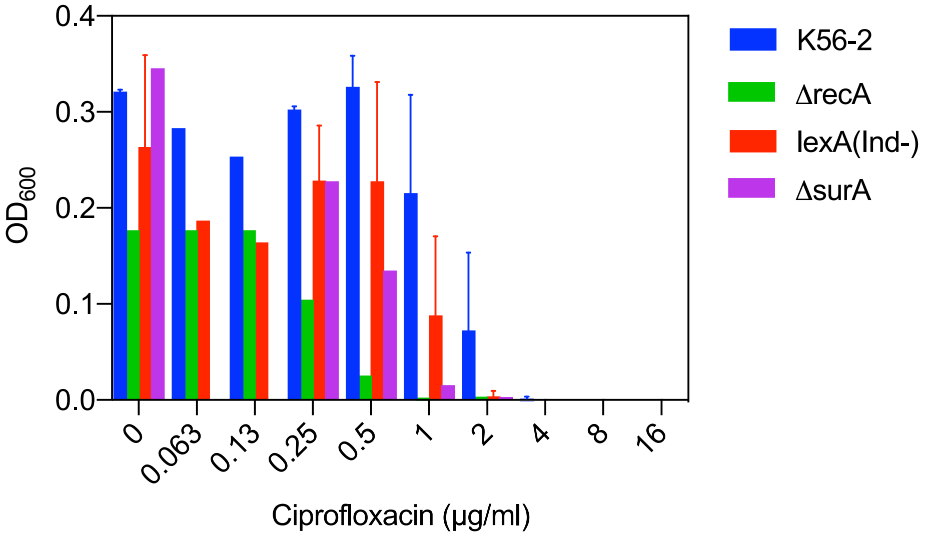

Supplementary References

1. Flannagan RS, Linn T, Valvano MA. A system for the construction of targeted unmarked gene deletions in the genus *Burkholderia*. *Environ Microbiol* 2008; **10**: 1652–1660.
2. Hogan AM, Jeffers KR, Palacios A, Cardona ST. Improved Dynamic Range of a Rhamnose-Inducible Promoter for Gene Expression in *Burkholderia* spp. *Appl Environ Microbiol* 2021; **87**.
3. Choi KH, Schweizer HP. mini-Tn7 insertion in bacteria with single attTn7 sites: Example *Pseudomonas aeruginosa*. *Nat Protoc* 2006; **1**.
4. Hogan AM, Rahman ASMZ, Lightly TJ, Cardona ST. A Broad-Host-Range CRISPRi Toolkit for Silencing Gene Expression in *Burkholderia*. *ACS Synth Biol* 2019; **8**: 2372–2384.
5. Lefebre MD, Valvano MA. Construction and Evaluation of Plasmid Vectors Optimized for Constitutive and Regulated Gene Expression in *Burkholderia cepacia* Complex Isolates. *Appl Environ Microbiol* 2002; **68**: 5956–5964.
6. Wayne P. Performance standards for antimicrobial susceptibility testing. 30th ed. CLSI supplement M100. Wayne, PA: Clinical and Laboratory Standards Institute. *Clsi M100 Ed30:2020* 2020.
7. Morón Á, Belinchón I, Tarhouchi AE, de Francisco P, Martín-González A, Amaro F. The transcriptome of *Burkholderia cenocepacia* reveals the activation of virulence factors and stress responses in protozoan phagosomes. In: Spanish Society for Microbiology (SEM) (ed). *XXIX Congress of the Spanish Society for Microbiology (SEM)*. 2023. Burgos, Spain.
8. Figurski DH, Helinski DR. Replication of an origin-containing derivative of plasmid RK2 dependent on a plasmid function provided in trans. *Proc Natl Acad Sci U S A* 1979; **76**: 1648-52.
